# The incredible shrinking puffin: Decreasing size and increasing proportional bill size of Atlantic puffins nesting at Machias Seal Island

**DOI:** 10.1371/journal.pone.0295946

**Published:** 2024-01-17

**Authors:** Heather L. Major, Joy E. Rivers, Quinn B. Carvey, Antony W. Diamond

**Affiliations:** 1 Department of Biological Sciences, Atlantic Laboratory for Avian Research, University of New Brunswick, Saint John NB, Canada; 2 Atlantic Laboratory for Avian Research, University of New Brunswick, Fredericton NB, Canada; University of Connecticut, UNITED STATES

## Abstract

Climate change imposes physiological constraints on organisms particularly through changing thermoregulatory requirements. Bergmann’s and Allen’s rules suggest that body size and the size of thermoregulatory structures differ between warm and cold locations, where body size decreases with temperature and thermoregulatory structures increase. However, phenotypic plastic responses to malnutrition during development can result in the same patterns while lacking fitness benefits. The Gulf of Maine (GOM), located at the southern end of the Labrador current, is warming faster than most of the world’s oceans, and many of the marine species that occupy these waters exist at the southern edge of their distributions including Atlantic puffins (*Fratercula arctica*; hereafter “puffin”). Monitoring of puffins in the GOM, at Machias Seal Island (MSI), has continued annually since 1995. We asked whether changes in adult puffin body size and the proportional size of bill to body have changed with observed rapid ocean warming. We found that the size of fledgling puffins is negatively related to sea surface temperature anomalies (warm conditions = small fledgers), adult puffin size is related to fledgling size (small fledgers = small adults), and adult puffins have decreased in size in recent years in response to malnutrition during development. We found an increase in the proportional size of bill to wing chord, likely in response to some mix of malnutrition during development and increasing air temperatures. Although studies have assessed clinal variation in seabird morphology with temperature, this is the first study addressing changes in seabird morphology in relation to ocean warming. Our results suggest that puffins nesting in the GOM have morphological plasticity that may help them acclimate to ocean warming.

## Introduction

Environmental changes often lead to physiological challenges as organisms struggle to meet their basic metabolic and thermoregulatory needs [1–4]. As a result, many organisms have experienced changes to their phenology and distribution resulting from climate change [5]. Warming, particularly in the marine realm, may have particularly acute impacts on ectotherms (e.g., crustacea, fish, squid) that seabirds eat, as many live close to their thermal maximum [6]; this can lead to cascading impacts up the food chain. In general, phenotypic plasticity allows organisms to meet changes in their physiological needs that can lead to changes in body size and allometry [but see 7,8]. In fact, decreasing body size has been termed the “third universal response to climate change” [9,10] and there are many documented accounts of decreasing body size with increasing temperature [e.g., 11–15]. Changing body size may be a result of a phenotypic plastic response to malnutrition during development [16], that leads to reduced growth rates and decreased adult size in many birds [17–19]. Alternatively, decreasing body size and allometric changes may be a genetic microevolutionary response to warming as explained by Bergmann’s and Allen’s rules. Bergmann’s Rule generalizes an increase in body size with decreasing temperature due to thermoregulatory benefits of decreased surface-area to volume ratio [20,21]. Allen’s Rule predicts larger appendage size in warmer locations due to thermoregulatory benefits of proportionally larger surface area over which to dissipate heat [22,23]. Allometric changes predicted by Allen’s Rule have been observed across many taxa and linked with climatic warming [24–26]. Regardless of the driver of change in body size and allometry, whether decreased adult size confers a fitness benefit will depend on whether environmental conditions remain favorable for the phenotype in question [19].

Seabirds are highly mobile, colonial nesting species that sit near the top of many marine food chains [27]. They are often used as indicators [28,29] or sentinels [30] for marine ecosystems and have shown many responses to ocean warming, including changes in phenology, foraging behaviours, and reproductive performance [31]. Many long-term, colony-based studies have documented changes in prey quality and quantity [e.g., 32,33], and decreases in nestling growth rate and fledgling size [34]. But because of the size of most seabird colonies, measuring individuals when they depart their natal site as fledgers and again as breeding adults is difficult. Similarly, it is not normally possible to age individuals accurately when they are captured as adults. Thus, we are unaware of any seabird study assessing phenotypic changes at the adult stage related to environmental conditions during development.

The Gulf of Maine (GOM) sits at the southern edge of many marine species ranges, including the copepod *Calanus finmarchicus* and Atlantic puffins (*Fratercula arctica*; hereafter “puffin”). The GOM is warming faster than most of the world’s oceans [35,36] and experienced a major regime shift in 2010, followed by numerous heat waves and changes in circulation that together resulted in a reduction in *Calanus* [37]. *Calanus* and other copepods are important prey items for many species of planktivorous fish (e.g., Atlantic herring *Clupea harengus* and American sandlance *Ammodytes americanus* [38,39]) that are important prey for seabirds and other top predators [40–42]. This decline of copepods was accompanied by decreases in herring abundance in both fisheries data and puffin chick diet [33,35]. As a result, puffin chicks in the GOM have experienced reduced growth rates, nestling survival, and size at fledging [31,33,43,44].

Machias Seal Island (MSI) is a small island located at the junction between the Bay of Fundy and the GOM and hosts the largest nesting colony of puffins in the GOM. MSI is the site of a long-term annual seabird monitoring program since 1995. This program includes an extensive capture-mark-recapture (CMR) program, in which both adults and chicks are captured/recaptured, measured, and released. A unique feature of MSI is the light station surrounded by a mown lawn where puffin fledglings congregate on the night of fledging and are easily captured and measured. This exceptional dataset records the size of fledgling puffins the night they depart the island for their first winter at sea and later measurements of these same individuals when they return as adults.

**Fig 1:**
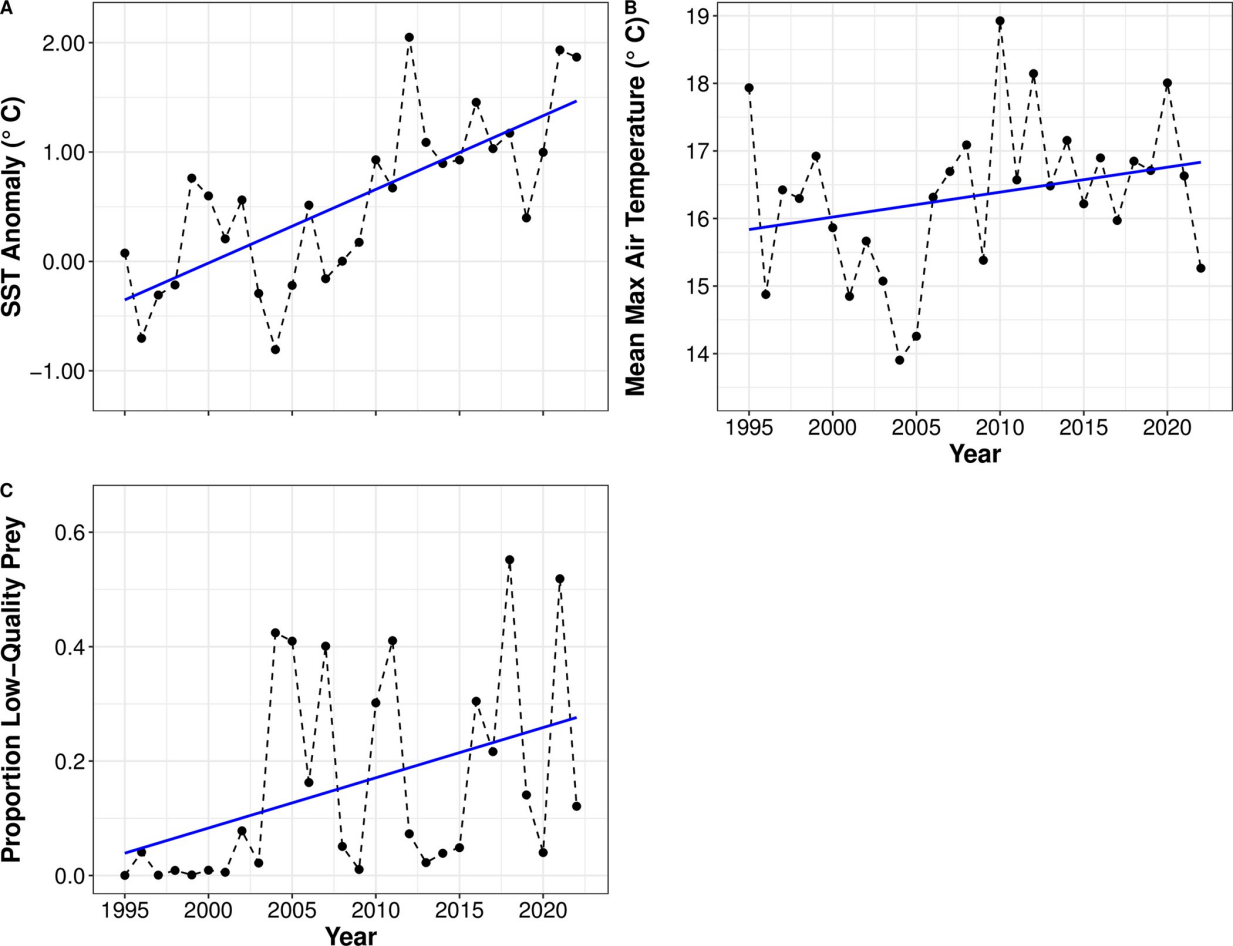
SST anomalies and proportion of high-quality prey in Atlantic puffin chick diets. Comparison of annual SST anomalies (A) in the Gulf of Maine, mean maximum air temperature in June and July (i.e., during chick rearing); B) and proportion of low-quality prey in chick diets (C) by year at Machias Seal Island during 1995–2022. Blue line is linear regression. High prey quality is defined as the percentage by biomass of Atlantic herring (*Clupeus harengus*) and sandlance (*Ammodytes hexapterus*) fed to chicks in each year [see 33].

Atlantic puffins are a small (~ 430 g), long-lived, pelagic seabird, their global nesting range extending from Spitsbergen, Norway and Novaya Zemlya, Russia, to the Gulf of Maine, USA at its southernmost extent [45]. At MSI, puffins return to the colony to begin breeding from three years after fledging, 96% have returned by age seven [43]. Puffins lay one egg each year and both adults incubate the egg and provision the chick. Puffin adult and chick diet at MSI is composed mainly of adult and larval sandlance, and juvenile white hake (*Urophycis tenuis*) and Atlantic herring [33]. Proportions of prey species fed to chicks can vary substantially both within and between years [31,33,46]. During years when chicks are nutritionally stressed (i.e., when prey quality, as characterized by Scopel et al. [33], and availability are low) chick growth rates and fledging size are reduced [31]. At MSI, as SST anomalies increase, the proportion of low-quality prey fed to chicks increases (see Fig 1), often resulting in slow chick growth rates and small size at fledging [44]. Puffin body size is indexed chiefly by wing chord length, and the puffin bill has recently been shown to dissipate heat [47] as is the case in many birds [48,49].

Our long-term dataset in the rapidly warming GOM provides an important opportunity to detect phenotypic changes in relation to ocean warming. Our study colony is among the furthest south of any regularly monitored site within the species’ range, in waters that are warming extremely rapidly, so puffins here are likely to be close to the limit of their thermal tolerance. Puffins are known to show considerable phenotypic plasticity in chick growth rates and fledging periods in relation to nesting habitat and associated vulnerability to predation [50,51] and food availability [52,53]. Our objective was to assess changes in adult body and bill size at MSI in relation to observed environmental conditions (i.e., warming and associated ecosystem changes) in the GOM (see Fig 1A). Here, we use sea surface temperature (SST) anomalies as a proxy of ecosystem-wide changes that have occurred in the GOM (i.e., reduced copepod and forage fish abundance and warming), proportion of low-quality prey fed to chicks as an indication of malnutrition during chick development, and mean maximum air temperature during chick rearing as an assessment of thermoregulatory environment during development. We note that changes in ambient temperature inside nesting burrows (where nestlings remain) may be buffered against the increases observed outside the burrows. We developed three competing hypotheses for change in body and bill size of Atlantic puffins 1) phenotypic plastic response to malnutrition during development; 2) genetic microevolutionary response to warming as explained by Bergmann’s and Allen’s rules; or 3) result of the interplay between malnutrition and genetic microevolutionary response to warming and resulting ecosystem-wide changes. When testing for a change in body size, we used a tiered approach; first, testing whether fledger body size has changed and is a function of environmental conditions (i.e., SST anomalies, prey quality fed to chicks, and maximum air temperature during chick rearing; see Fig 1). We then asked if fledger body size predicts adult body size, and finally whether observed changes in adult body size are the result of malnutrition, warming, or some combination of both. We predict that change in body size is a phenotypic plastic response to malnutrition during development, as suggested by previous studies [31,33,46]. Avian bills are highly vascularized, poorly insulated, and used for thermoregulation by many species [48,49] including puffins [47], and given the heat-dispersion hypothesis embedded in Allen’s rule, we predict that a warming environment increases the demand for heat dissipation and larger thermoregulatory structures would be selected for during development. Similar to body size, many birds show clines in bill size [see review in 23,48], changes in bill size in relation to warming have been documented for many bird species [24,25], though not, to our knowledge, for seabirds. We predict that if puffins use their bills for thermoregulation, adult bill size will increase in proportion to body size as temperatures increase, as predicted by Allen’s Rule.

## Materials and methods

### Study site

Machias Seal Island (MSI) is a small island (~9.5 hectares) located at the mouth of the Bay of Fundy and the edge of the GOM that is designated as a migratory bird sanctuary. The seabird nesting colony at MSI includes nesting populations of Atlantic puffin, razorbill (*Alca torda*), common murre (*Uria aalge*), Arctic tern (*Sterna paradisaea*), common tern (*S*. *hirundo*), Leach’s storm-petrel (*Hydrobates leucorhous*), and common eider (*Somateria mollissima*). Members from the Atlantic Laboratory for Avian Research (ALAR) at the University of New Brunswick have been studying the seabird populations at MSI since 1995. Beginning in 1980, personnel from the Canadian Wildlife Service banded and resighted puffins at MSI, but field work at MSI before 1995 was not annual. All research conducted at MSI since 1995 has occurred under approved annual animal use protocols from the University of New Brunswick, and annual scientific, migratory bird sanctuary, and bird banding permits from Environment and Climate Change Canada. All data included in our analyses were collected as part of the long-term monitoring program at MSI during 1995–2022, methods are detailed in island protocols that are available online: https://msialar.wixsite.com/alar-msi/msi-protocols.

Our analyses rely on data collected for individuals at both the fledger and adult stage, because individuals begin attending the colony in their third to fifth years after fledging and recapture rates of previous banded adults is small (~18% of our captures each year are previously banded individuals), sample sizes of adults banded as fledgers (~4% of our captures each year are adults that were banded as fledgers) decreases rapidly for birds that fledged in the most recent ten to fifteen years and is < five after 2011 and zero after 2015. Due to this limitation and limited sex information (see below) our sample sizes and the fledge years included vary across our four main analyses.

### Data collection

#### Lighthouse fledger body size

Beginning in mid- to late-July each year, puffin fledglings depart MSI at night for the non-breeding season when they remain at sea. Many are attracted to the light from the light station located near the middle of MSI and congregate on the mown lawn in front of it. Every half hour during the hours of darkness, ALAR researchers patrol the lawn checking for puffin fledgers. Each fledger encountered is captured by hand, banded with a Bird Banding Laboratory (BBL) band and an alpha-numeric field readable band (FRB), measured (mass [grams], natural wing chord length [mm], culmen [mm], and head + bill [mm]), and then released into the water.

#### Banding and recapture

May through August of each year, adult puffins are captured at their nest sites, by grubbing nesting burrows and using box traps (a trap placed in the colony with a swivel-top lid that when a puffin lands on the surface deposits the bird into the box and closes again). All birds captured are banded with a BBL and FRB, measured (mass [grams], natural wing chord length [mm], culmen [mm], bill depth [mm], and head + bill [mm]), and released. Birds that were previously banded are measured as above, bands read, and released.

As part of a larger monitoring program on MSI, approximately 70–100 puffin nesting burrows are monitored each year to measure reproductive success. All chicks in these burrows that survive to 30–35 days are banded with a BBL and FRB. For burrow-banded puffin chicks and lighthouse fledgers, hatch year (here termed “fledge year”) is known. When one of these birds is recaptured as an adult, its age in years is known, and these individuals are included in our sample of known-age adults. For our analyses, we consider only individuals that were banded as chicks and recaptured as adults (hereafter termed “known-age”).

#### Sex determination

Male and female puffins cannot be distinguished visually; we used two methods, genetic sex determination and discriminant function analysis, to determine the sex of the individuals in this study. During banding, 5–6 breast feathers are plucked for genetic analysis. Over the years different ALAR researchers have conducted genetic analyses for puffins on MSI for different projects. We collated that information and have genetic sex information for a total of 1,529 individual puffins. In all cases, we used the methods outlined in Fridolfsson and Ellegren [54] Devlin et al. [55], and Friars and Diamond [56]. For individuals that did not have genetic sex information we used the discriminant functions developed by Friars and Diamond [56], using bill length and depth. Specifically, we used equations 2 (for birds with fewer than 1.5 bill grooves or no bill groove information) and 4 (for older birds with more than 1.5 bill grooves) to assign individuals as male or female. Our approach for sex determination was conservative; for equation 2 we assumed birds were female if the discriminant score was < -0.612, or male if the discriminant score was > 0.960, all other birds were classified as unknown. For equation 4 we assumed birds were female if the discriminant score < -0.605, or male if the discriminant score was > 0.838, again all other birds were classified as unknown. To test the efficacy of the discriminant function, we applied it to all birds with genetic sex information and calculated the proportion of those birds that were designated male or female by both methods and those that were not. Assuming that the genetic analysis was the correct sex designation, we found that 85% (88% correct assignments for females and 83% correct assignments for males) of the individuals were assigned the same sex using both methods, providing support for including birds sexed using the discriminant function.

### Environmental conditions

SST data was downloaded from the National Oceanic and Atmospheric Administration (NOAA) Physical Sciences Laboratory website (https://www.psl.noaa.gov). We extracted SST monthly mean data from the NOAA Optimum Interpolation (OI) SST V2 high resolution dataset for all years between January 1, 1981 –December 31, 2022 for 18 1°×1° cells that lie within the GOM, which encompasses the full extent of puffin foraging range during the breeding season [57,58]. We calculated annual means for each year in our dataset and an overall mean, to use as our reference period, for the period January 1, 1981 –December 31, 2011. Anomalies were then calculated as the difference between the annual mean and the reference period.

Air temperature (degrees Celsius; daily min and max) is recorded every day during the puffin nesting season at 2100 h Atlantic Daylight time (ADT). Here, we calculated the mean maximum air temperature for June 1 –July 31 in each year and used that as an index of air temperature during chick development.

Prey quality, or the proportion of low-quality prey fed to chicks, is calculated as a proportion of the total biomass of prey considered “low-quality” [see 33] recorded during multiple 3-hour prey watches that occur each year during chick rearing. For detailed methods see [33,59].

### Statistical analyses

All statistical analyses were completed in the RStudio environment (R version 4.2.0 [60,61]) using an information theoretic approach. Specifically, we used the R package “*MuMIn*” [62] and ranked models using Akaike’s Information Criterion (AIC) for small sample sizes (AICc), and AICc weights were used to evaluate model likelihood [63]. We used parameter estimates and standard errors to draw inference from our data. When the top model received less than 90% of the total weight among models, we used model averaging (from the “*MuMIn*” package) and the “conditional average” method [see 63,64]. We then used weighted parameter estimates and unconditional standard errors to draw inference, as above. When testing among models that include SST anomaly, mean maximum air temperature, and prey quality, we standardized our effect sizes on two standard deviations following Gelman [65]. Summary data are presented as means ± 95% CI, unless otherwise noted.

### Change in lighthouse fledger size

To evaluate whether lighthouse fledger size (i.e., wing chord length) is correlated with environmental conditions, we used the “*nlme*” package in R [66] and generalized linear mixed models with a gaussian distribution. Lighthouse fledger data included all fledgers captured on the mown lawn in front of the light station during 1995–2019 that were of known sex (*n* = 1,020). Male puffins average slightly larger than females in all dimensions [53,56] and we expect annual variation, thus our candidate models all had Sex and Year included as random effects. First, we ran the most parameterized model (i.e., global model) fit by restricted maximum likelihood (REML) and checked model diagnostics. Our diagnostics revealed assumptions of linearity were met but temporal autocorrelation was present. We defined autocorrelation structure using autoregressive moving average (ARMA) serial correlation structure. To find the optimal ARMA structure, we ran models with all combinations of *p* and *q* between zero and three and compared them with AIC. The model with the lowest AIC value was taken as the model containing the optimal ARMA structure (see [67] for more information); that ARMA structure was then incorporated into our *a priori* candidate model set that was run as above but fit with maximum likelihood (ML).

### Change in adult body size

#### Relationship between fledger and adult body size

To assess the relationship between fledger and adult body size (i.e., whether a small fledger becomes a small adult) we ran a series of linear regressions with no autocorrelation structure evaluating the relationship between structural measurements (i.e., wing chord length, culmen, and head+bill) taken from a lighthouse fledger and from the same individual captured as an adult, following the same AIC procedure detailed above but including only Sex as a random term. Our dataset included all individual puffins that were measured as both fledgers and adults from fledge years 1995–2015, and that have sex information (*n* = 285). In some instances (*n* = 54) individual adults were captured and measured in multiple years; here, we used the average of all measurements taken for that individual, so each individual is represented only once.

#### Change in adult size

Using all known-age adults with measurements, we tested whether aspects of adult puffin body size (i.e., wing chord length, head+bill length, and proportional bill size to wing chord length) have changed as a function of environmental conditions during their fledge year. We used two indices to represent bill size: bill depth and bill area, treating the bill as an isosceles triangle and using the following equation for the area of a triangle:

BillArea=0.5×(Culmen2−(BillDepth2)2×BillDepth)

We used the same statistical procedures as outlined above for lighthouse fledgers. This analysis is a direct test assessing 1) reduction in the structural size of adult puffins and 2) increase in proportional bill size to body size. Here our dataset includes data for all adults banded as both lighthouse fledgers and as chicks prior to fledging, and that have sex information for years 1995–2011 (*n* = 286), as our sample size for known-age adults with measurements for fledge years after 2011 is low (< 5) and reaches zero by fledge year 2016. Similar to our dataset assessing correlation between fledger and adult size, if an individual was captured and measured more than once, we used the average of all measurements taken for that individual, so each individual is represented only once.

## Results

### Environmental conditions

SST anomaly varied with year between 1995–2022, ranging between -0.81 to 2.05 (Fig 1A). All anomalies higher than 0.92 occurred after 2009. Similarly, mean maximum air temperature and the proportion of low-quality prey fed to chicks varied with year and ranged between 13.4°C– 18.9°C (Fig 1B) and 0.0002–0.55 (Fig 1C). Years with mean maximum air temperature > 18.0°C have all occurred after 2010, years with the highest proportion of low-quality prey fed to chicks (i.e., > 0.40) all occurred after 2004.

### Change in lighthouse fledger size

**Fig 2:**
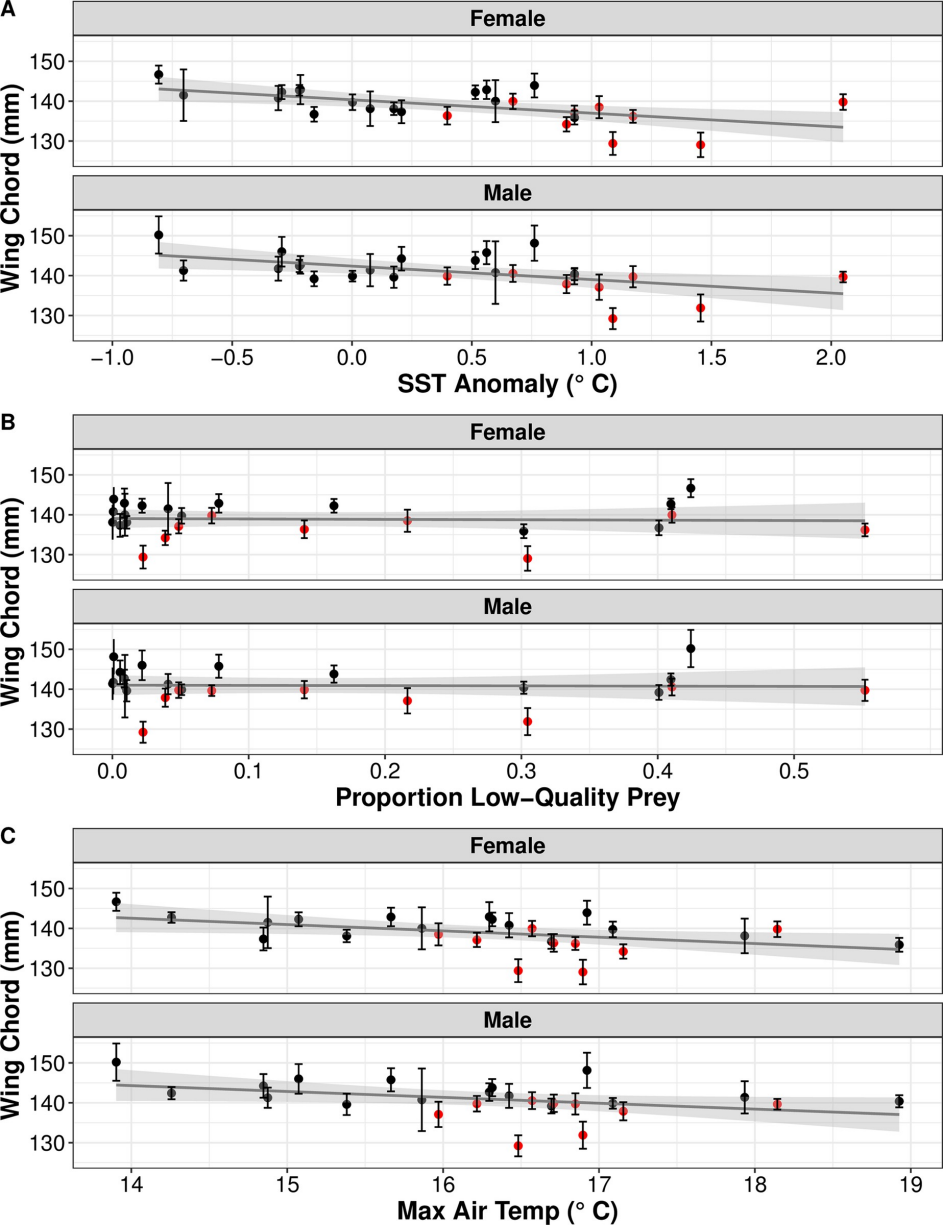
Atlantic puffin lighthouse fledger wing chord length and environmental conditions. Correlation between mean (± 95% CI) Atlantic puffin lighthouse fledger wing chord length at Machias Seal Island during 1995–2019 and (A) annual SST anomalies in the Gulf of Maine (SST anomalies were calculated using 1981–2011 as the reference period), (B) proportion of low-quality prey fed to chicks, and (C) mean maximum air temperature during chick development for females and males. Data from 2011–2019 are in red.

Between 1995–2022 we have measurement data for 5,348 lighthouse fledgers (average 163 ± 35 individuals measured per year). Of those, we have sex designation for 1,020 individuals (505 female and 515 male). Male lighthouse fledgers (wing chord length 140 ± 0.56 mm) tend to be larger than females (138 ± 0.55 mm), and on average we have observed a decrease in the size of both males and females as SST anomalies, mean maximum air temperature, and proportion of low-quality prey fed to chicks all increase (Fig 2). We did not include data from before 1995 (the start of our standardized protocols), but there are on average 94 ± 30 (range 17–192) lighthouse fledger measurements per year dating back to 1980 (no measurement data from 1985–1987). We have very little sex information for those birds, but in general, mean fledger wing chord length between 1980–1994 was 142 ± 0.17 mm, compared with 138 ± 0.29 mm between 2006–2022.

Our top candidate model, included the term SST anomaly, received 96% of the weight among our candidate models (Table 1). Our model averaged parameter estimates and standard errors suggest an important negative correlation between annual SST anomalies and fledger wing chord (Table 2). Our parameter estimates and unconditional standard errors also suggest a weak negative correlation between fledger body size and mean maximum air temperature but no discernable relationship with prey quality (Table 2).

**Table 1:** Candidate models assessing correlation between Atlantic puffin lighthouse fledger wing chord length (WC) and environmental conditions at Machias Seal Island during 1995–2019. All candidate models included autoregressive moving average autocorrelation structure *p* = 1, *q* = 1, and the random terms Sex and Fledge Year (*n* = 1,020).

Model	k	logLik	AICc	delta	weight
WC = SST Anomaly	7	-3160.45	6335.01	0.00	0.96
WC = Max Air Temp	7	-3163.79	6341.69	6.67	0.03
NULL	6	-3167.99	6348.06	13.05	0.00
WC = Prey Quality	7	-3167.87	6349.84	14.83	0.00

**Table 2:** Parameter estimates, standard errors, and relative likelihoods for the top supported candidate model evaluating difference in Atlantic puffin lighthouse fledger wing chord length (WC) and annual SST anomalies between 1995–2019 at Machias Seal Island. Parameters in bold font do not bound zero.

	Parameter Estimate	Unconditional Standard Error	Relative Likelihood
Intercept	139.27	0.64	
**SST Anomaly**	**-2.40**	**0.58**	**0.96**
**Max Air Temp**	**-0.67**	**0.22**	**0.03**
Prey Quality	-5.05	10.09	0.00

### Change in adult body size

#### Relationship between fledger and adult body size

**Fig 3:**
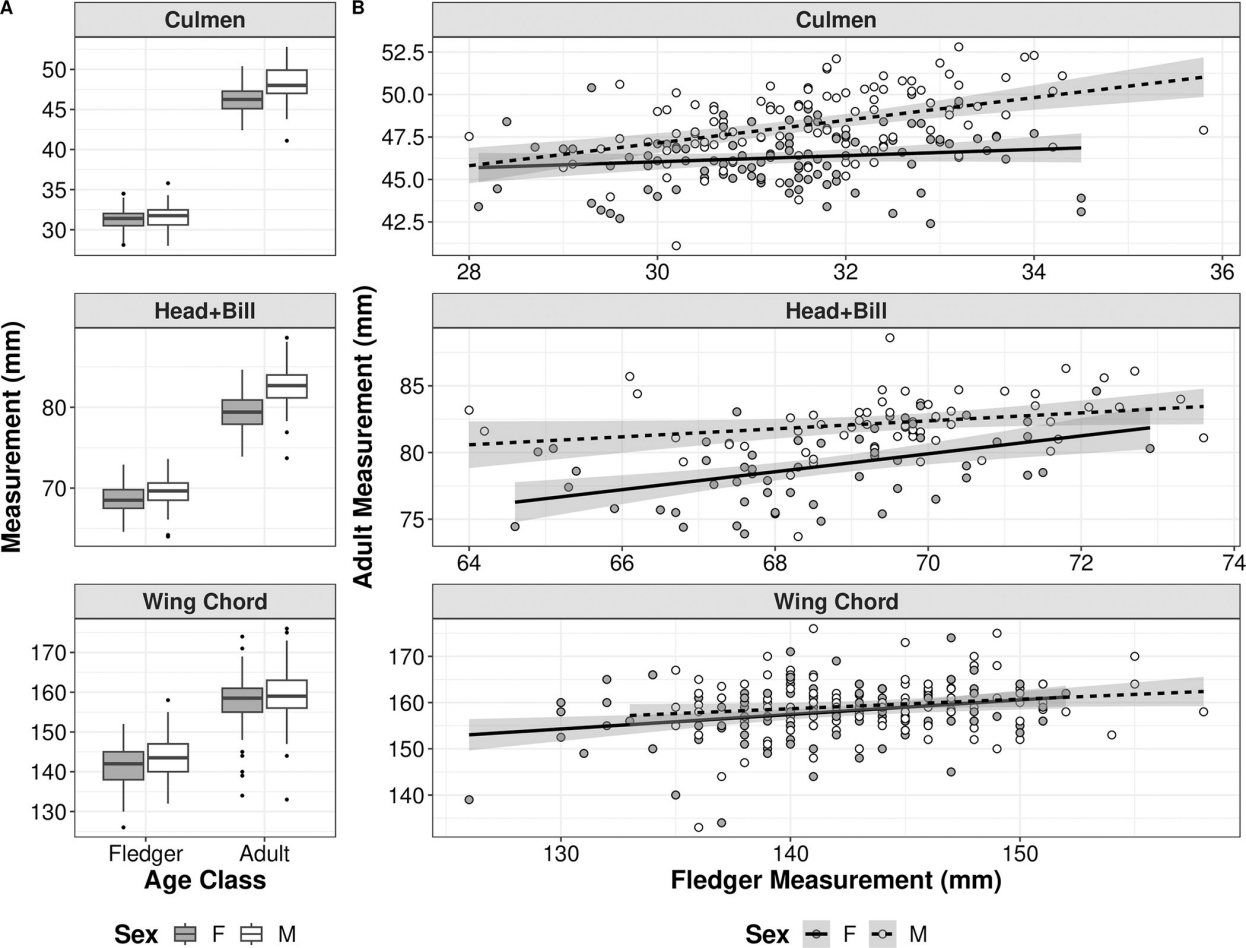
Size difference and correlation between Atlantic puffin fledgers and adults. Comparison of (A) difference in size at the fledger and adult stage and (B) correlations between adult and fledger Atlantic puffin (male [white] and female [dark grey]) culmen, head+bill, and wing chord lengths at Machias Seal Island between 1995–2015. Box plots in (A) show the median (black line), first and third quartiles, and the whiskers extend to 1.5 interquartile range (IQR), the black dots are outliers; lines in (B) are linear regressions with 95% confidence intervals.

At MSI, a total of 285 known-age adult puffins were measured at least once after being measured as a lighthouse fledger. Measurements of culmen and head+bill were not as consistently recorded as wing chord measurements, particularly on fledgers; sample sizes for known-sex culmen, head+bill, and wing chord are 221 (111 male, 110 female), 122 (63 male, 59 female), and 251 (129 male, 122 female), respectively. In all cases, average measurements of structural size were smaller for fledgers than for adults and for females than for males (Fig 3A). Adult culmen, head+bill, and wing chord all show a general trend suggesting a positive correlation between fledger and adult size (Fig 3B). Our statistical analyses support this trend for all three variables and show positive correlations between each set of measurements (Tables 3 and 4).

**Table 3:** Candidate models assessing whether Atlantic puffin adult culmen (CUL), head+bill (HB), and wing chord (WC) are a function of fledgling culmen, head+bill, and wing chord length. All data are from Machias Seal Island, 1995–2015, the term Sex is included as a random effect in all models.

Model	k	logLik	AICc	delta	weight
**Culmen** (*n* = 221)					
Adult CUL = Chick CUL	4	-448.15	904.49	0.00	1.00
NULL	3	-459.68	925.47	20.98	0.00
**Head+Bill** (*n* = 122):					
Adult HB = Chick HB	4	-278.57	565.49	0.00	1.00
NULL	3	-287.44	581.09	15.60	0.00
**Wing Chord** (*n* = 251):					
Adult WC = Chick WC	4	-797.25	1602.66	0.00	1.00
NULL	3	-804.47	1615.03	12.37	0.00

**Table 4:** Model averaged parameter estimates, unconditional standard errors, and relative likelihoods for the candidate model sets evaluating the relationship between Atlantic puffin adult and fledger culmen (CUL), head+bill (HB), and wing chord (WC) at Machias Seal Island during 1995–2015. Parameters in bold font are those that do not bound zero.

	Parameter Estimate	Unconditional Standard Error	Relative Likelihood
**Culmen:**			
Intercept	33.06	2.97	
**Chick CUL**	**0.45**	**0.09**	**1.00**
**Head+Bill:**			
Intercept	47.02	7.82	
**Chick HB**	**0.49**	**0.11**	**1.00**
**Wing Chord:**			
Intercept	120.18	10.12	
**Chick WC**	**0.27**	**0.07**	**1.00**

#### Change in adult size–Wing chord length and Head+Bill length

**Fig 4:**
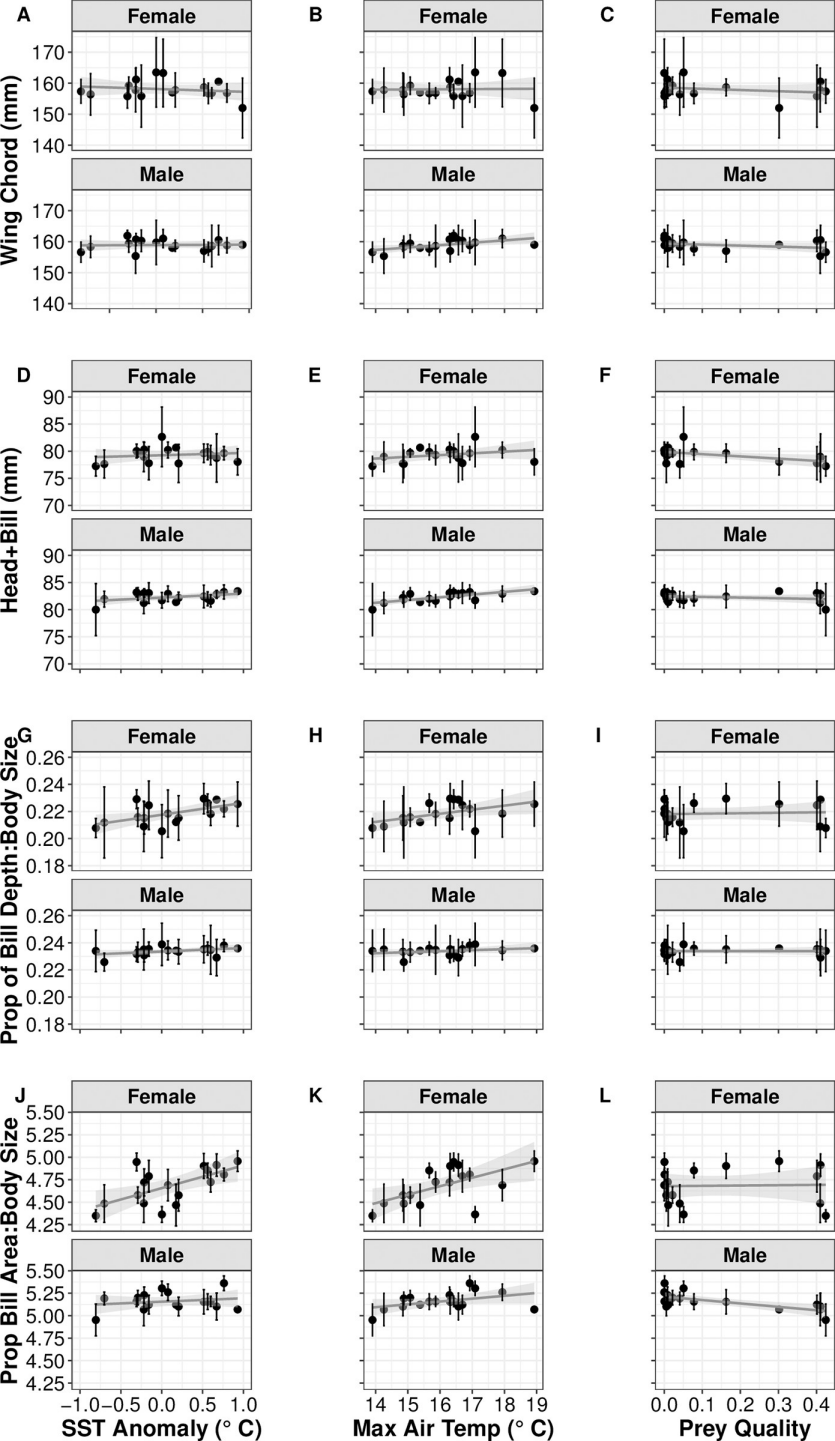
Difference in structural size of male and female Atlantic puffins in relation to environmental conditions. Comparison of wing chord (A, B, & C), head+bill (D, E, & F), and proportional size of bill depth to body size (G, H, & I) and bill area to body size (J, K, & L) of male and female known age Atlantic puffins (*Fratercula arctica*) from Machias Seal Island in relation to SST anomaly (A, D, G, & J), Mean maximum air temperature (B, E, H, & K), and proportion of low-quality prey (Prey Quality; C, F, I, & J) in the year of fledging during 1995–2011.

Over our 28-year time series at MSI we have measured 363 adults of known age (153 female, 165 male, and 45 of unknown sex). We note variability in the number of individuals measured in each fledge year, here we included only birds from fledge years between 1995–2011 (*n* = 276; 132 female, 144 male). The total number of individuals measured as adults from each of these fledge years averaged 25 ± 7. We note variability in changes in size of adult wing chord and head+bill with our different environmental conditions, ranging from no discernable change, to both positive and negative changes (Fig 4A–4F). Our statistical analyses show a negative relationship between both wing chord and head+bill, and proportion of low-quality prey (Tables 5 and 6). We also note weak positive relationships between both structures and mean maximum air temperature, and negative (wing chord) and positive (head+bill) relationships with SST anomaly (Table 6).

**Table 5:** Candidate models assessing whether Atlantic puffin adult head+bill (HB), wing chord (WC), ratio between bill depth and wing chord (BD:WC), and the ratio between bill area and wing chord (BA:WC) are a function of environmental conditions. All data are from Machias Seal Island, 1995–2011, the terms Sex and Fledge Year were included as random effects in all models.

Model	k	logLik	AICc	delta	weight
**Head+Bill** (*n* = 273):					
Adult HB = Prey Quality	5	-614.71	1239.64	0.00	0.97
Adult HB = Mean Max Air Temp	5	-618.21	1246.65	7.01	0.03
NULL	4	-621.60	1251.34	11.71	0.00
Adult HB = SST Anomaly	5	-620.83	1251.89	12.25	0.00
**Wing Chord** (*n* = 274):					
Adult WC = Prey Quality	5	-877.43	1765.07	0.00	0.53
NULL	4	-879.47	1767.08	2.01	0.19
Adult WC = Mean Max Air Temp	5	-878.66	1767.54	2.47	0.15
Adult WC = SST Anomaly	5	-878.90	1768.03	2.96	0.12
**Bill Depth: Wing Chord** (*n* = 268):					
Adult BD:WC = SST Anomaly	5	765.80	-1521.37	0.00	0.92
Adult BD:WC = Mean Max Air Temp	5	762.77	-1515.32	6.05	0.04
NULL	4	761.22	-1514.30	7.07	0.03
Adult BD:WC = Prey Quality	5	761.24	-1512.25	9.11	0.01
**Bill Area: Wing Chord** (*n* = 267):					
Adult BA:WC = Mean Max Air Temp	5	-121.48	253.19	0.00	0.73
Adult BA:WC = SST Anomaly	5	-122.58	255.38	2.19	0.24
Adult BA:WC = Prey Quality	5	-125.09	260.42	7.23	0.02
NULL	4	-127.01	262.18	8.99	0.01

**Table 6:** Model averaged parameter estimates, unconditional standard errors, and relative likelihoods for the candidate model sets evaluating the relationship between Atlantic puffin adult head+bill, wing chord, ratio between bill depth and wing chord, and ratio between bill area and wing chord, and SST anomalies at Machias Seal Island during 1995–2011. Parameters in bold font are those that do not bound zero.

	Parameter Estimate	Unconditional Standard Error	Relative Likelihood
**Head+Bill:**			
Intercept	80.94	1.21	
**Mean Max Air Temp**	**0.18**	**0.07**	**0.03**
**SST Anomaly**	**0.45**	**0.37**	**0.00**
**Prey Quality**	**-12.60**	**3.02**	**0.97**
**Wing Chord:**			
Intercept	158.72	0.46	
**Mean Max Air Temp**	**0.22**	**0.17**	**0.15**
**SST Anomaly**	**-0.92**	**0.84**	**0.12**
**Prey Quality**	**-17.55**	**8.29**	**0.53**
**Bill Depth:Wing Chord:**			
Intercept	0.23	0.01	
Mean Max Air Temp	0.00	0.00	0.04
**SST Anomaly**	**0.01**	**0.00**	**0.92**
Prey Quality	-0.00	0.02	0.01
**Bill Area:Wing Chord:**			
Intercept	4.95	0.18	
**Mean Max Air Temp**	**0.03**	**0.01**	**0.73**
**SST Anomaly**	**0.15**	**0.05**	**0.24**
**Prey Quality**	**-1.01**	**0.51**	**0.02**

### Change in adult size–Proportional bill size

On average, the proportional size of bill depth to body size (i.e., wing chord length) was 0.23 ± 0.002 (range 0.18–0.29). Overall, we see a trend towards a larger proportional size (for both proportional bill size and proportional bill area) in years with higher SST anomalies (Fig 4G and 4H) and mean maximum air temperature (Fig 4J and 4K). We observe little change in proportional bill size with proportion of low-quality prey (but note negative relationship for male bill area; Fig 4I and 4L). We note that the top candidate model for these two metrics of proportional bill size differ, with SST anomaly being the top supported model for proportional bill size and the top supported model for proportional bill area being mean maximum air temperature (Table 5). We also note weak positive and negative support for SST anomaly and proportion of low-quality prey, respectively, for proportional bill area (Table 6).

## Discussion

Both Bergmann’s and Allen’s rules predict that body/appendage size is mediated by temperature; with warming, body size should decrease (Bergmann’s rule) and thermoregulatory structures should increase in proportion to body size (Allen’s rule), but changes to body size can also be the result of malnutrition during development with no genetic microevolutionary change. We developed three competing hypotheses to explain observed changes in body and bill size of Atlantic puffins at Machias Seal Island, where rapid ocean warming and changes in prey quality are occurring: 1) phenotypic plastic responses to malnutrition during development; 2) genetic microevolutionary responses to warming as explained by Bergmann’s and Allen’s rules; and 3) result of the interplay between malnutrition and genetic microevolutionary responses to warming from ecosystem-wide changes. We did not find consistent support for any single hypothesis solely. We found that fledger body size is related to SST anomaly, supporting our third hypothesis; and that fledger and adult body and bill size are related. Thus, we would predict that adult body size should also be related to SST anomalies, but our analysis for adult body size found relatively strong support for our first hypothesis, suggesting that even though ecosystem-wide changes are most important for fledger size, the proportion of low-quality prey fed to chicks prior to fledging is most important for determining adult body size. Further, we found that proportional bill size/area support hypotheses 2) and 3), depending on the metric used. In both cases proportional bill size is larger with warming (i.e., SST and mean maximum air temperature). The differences among structures is intriguing and may suggest that we did not include an important predictor of adult size, or that our third hypothesis (some combination of hypotheses 1 and 2) is the best explanation for changes in puffin body and bill size. We note that responses of male and female puffins differed, and females seem to generally have a stronger response to warming than males, particularly in proportional bill size. A *post hoc* test conducted on each sex separately, shows variability in responses to environmental conditions by females and males for each structure measured (see S1 and S2 Tables). For proportional bill size we found that SST anomaly is important for both sexes, but for proportional bill area, prey quality is most important for males and mean maximum air temperature for females (S1 and S2 Tables). We postulate that differences may result from the different energetic demands faced by males and females, particularly during egg production, but future work is needed to investigate these differences.

Atlantic puffins are considered a cold-adapted species, their global nesting range extending from Spitsbergen, Norway and Novaya Zemlya, Russia, to the Gulf of Maine, USA at its southernmost extent [45]. A cline in body size has been documented, with the largest puffins (with largest bill) nesting in the coldest regions [45,68], and among the different puffin sub-species, the largest, *F*. *arctica naumanni*, nesting in the high Arctic and the smallest, *F*. *arctica grabae*, nesting in the southeast Atlantic (those nesting in the GOM (*F*. *a*. *arctica*) are intermediate in size [53,68]). Although puffins with the largest bills nest in the most northern (i.e., coldest) regions and Lowther et al. [45] state that proportionally the *naumanni* sub-species has the largest bill, evidence from published accounts of morphometrics suggest this is not the case (see S3 and S4 Tables, and S1 and S2 Figs). Rather a cline exists where the proportional size of the bill to body is largest at the most southern colonies where the *grabae* and *arctica* sub-species nest. Thus, variability in body and bill size is present, and given differences in metabolic and thermoregulatory requirements in different regions, local acclimation is expected. Our results support that local acclimation is occurring in Atlantic puffins nesting in the GOM and may help them adapt to the rapidly warming environment. We note that we would expect proportional bill size to be larger if body size is decreasing and bill size remains the same. To test, *post hoc*, whether the change in proportional bill size is an artefact of changing body size, we assessed whether absolute bill size followed the same trends as proportional bill size. Here, we found the relationship holds and that mean maximum air temperature is positively related to absolute bill size (i.e., absolute bill size and proportional bill area both increase with increasing air temperatures; S5 and S6 Tables), lending support to our conclusion that proportional bill size is changing with environmental conditions.

Our results are consistent with both a phenotypic plastic response and genetic microevolutionary changes with warming. Van Gils et al. [16] suggest that trophic disruptions that result in a size change are separate from Bergmann’s and Allen’s rules and we have not tested for a genetic change or whether observed changes are adaptive. We suggest that ecosystem-wide changes resulting from warming can lead to variable responses and that these two rules are not mutually exclusive. We postulate that changing size of puffins nesting at MSI is the result of both trophic disruption and warming. Further research is necessary to understand if the changes we have observed confer a fitness benefit and whether malnutrition, as the proximate cause of change in size, can lead to an adult that is better adapted to their warming environment.

All individual puffins measured were larger in the adult stage than as fledgers, providing evidence of continued growth post fledge and Georgantopoulos [44] found that large fledgers at MSI grew less after fledging than smaller fledgers, suggesting compensatory growth. The impact of compensatory growth often means lower survival, as individuals allocate energy to growth instead of to improving their condition [69]. A study of tufted puffins (*Fratercula cirrhata*) at Triangle Island, BC found that small fledgers are less likely to return to the island as adults than large fledgers [70]; if the same is true for Atlantic puffins at MSI, our sample is biased against finding what we predicted.

Our finding of smaller bodies and larger bills for individuals that fledged during years where environmental conditions were warmer/prey quality was low is particularly interesting given that our analysis did not include the most recent years when all environmental variables are highest. This is particularly true for proportional bill size/area. If puffin bills are used as thermoregulatory structures and their size is determined during development, it is possible that phenotypic plasticity and the ability to alter the size of the bill depending on environmental conditions during development may be crucial in the long-term persistence of puffins in the GOM. However, much research is needed to fully understand the importance of bills as thermoregulatory organs, how bill size is determined, and the phenotypic limits of variability in this trait.

Puffins nesting in the GOM are experiencing some of the most rapid warming occurring around the globe. Documented changes to the quality of food fed to chicks, and decreases in chick growth rate, fledgling body condition, and reproductive success [31,33,46] suggest that environmental changes are negatively affecting this population. To date, we have not observed a decrease in the nesting population of Atlantic puffins at MSI, or a decrease in annual survival (although research updating our survival estimates is ongoing) suggesting that, for now, puffins have been able to cope with warming. Exactly how they are coping, the mechanisms involved, and the fitness consequences remain unknown. Here, we have used our MSI dataset of measurements of known-age puffins to complete the first assessment of seabird responses to climate change with respect to the malnutrition hypothesis, and Bergmann’s and Allen’s rules. Further research is needed to assess whether the changes we have observed confer a fitness benefit and are heritable.

Nutritional stress during development, particularly during the period of adult provisioning, has been shown to result in reduced growth rates and decreased adult skeletal size in song sparrows (*Melospiza melodia*) and other birds [17–19,71]. In a study of growth allocation of Atlantic puffin chicks, Øyan and Anker-Nilssen [72] found that when stressed for food, Atlantic puffin chicks allocate more energy to growing their head (including their bill) over that of their wings. Thus, during years of reduced prey quality we would expect puffin wings to be smaller at fledge and bills to be proportionally larger, as our results show. However, we would not expect to see an absolute increase in bill size as observed, rather a proportional increase resulting from smaller wings. Assuming thermoregulatory constraints are an important component of Atlantic puffin fitness, we hypothesize that the fitness benefits conferred result in higher survival and recruitment rates for individuals with larger bills and smaller bodies. Thus, the impact of nutritional stress on Atlantic puffin chicks on MSI may help the population adapt to rapid warming in the GOM. However, future work is required to assess the mechanistic processes responsible for determining body size and allometry, how these processes change in relation to nutritional stress and/or changing environmental conditions, and whether the observed changes in body and proportional bill size confer a fitness benefit.

## Supporting information

S1 Fig Mean monthly Sea Surface Temperature (SST;°C) for the North Atlantic and Arctic oceans for July 2000. Map generated from NOAA OI SST V2 High Resolution Dataset (https://psl.noaa.gov/mddb2/makePlot.html?variableID=156646). Arrows denote approximate location of each colony.
(DOCX)

S2 Fig Proportional bill size of the three Atlantic puffin (*Fratercula arctica*) subspecies (*F*.*a*. *arctica*, *grabae*, *and naumanni*) over their North Atlantic distribution. Data collated from A) Table 2 in Burnham et al. 2020 [68] and B) Appendix 1 in Harris and Wanless 2011 [53].
(DOCX)

S1 Table Candidate models assessing whether male and female Atlantic puffin adult head+bill (HB), wing chord (WC), ratio between bill depth and wing chord (BD:WC), and the ratio between bill area and wing chord (BA:WC) are a function of SST anomaly, mean maximum air temperature, and prey quality fed to chicks. All data are from Machias Seal Island, 1995–2011. Models were general linear models run using the “*nlme*” R package in the RStudio environment and include the term Fledge Year as a random effect.
(DOCX)

S2 Table Model averaged parameter estimates, unconditional standard errors, and relative likelihoods for the candidate model sets evaluating the relationship between male and female Atlantic puffin adult head+bill, wing chord, ratio between bill depth and wing chord, and ratio between bill area and wing chord, and SST anomalies at Machias Seal Island during 1995–2011. Parameters in bold font are those that do not bound zero. Model averaging was completed using the “*MuMIn*” R package in the RStudio environment.
(DOCX)

S3 Table Comparison of proportional bill size (culmen length) to wing chord length of Atlantic puffins (*Fratercula actica*). Data from Table 2 Burnham et al. 2020, sea surface temperature (SST) estimated from NOAA OI SST V2 High Resolution dataset for July 2020 (see S1 Fig).
(DOCX)

S4 Table Comparison of proportional bill size (straight bill length) to wing chord length of Atlantic puffins (*Fratercula arctica*). Data from Appendix 1 Harris and Wanless 2011; sea surface temperature (SST) estimated from NOAA OI SST V2 High Resolution dataset for July 2020 (see Fig 1).
(DOCX)

S5 Table Candidate models assessing whether male and female Atlantic puffin bill depth (BD) is a function of SST anomaly, mean maximum air temperature, and prey quality fed to chicks. All data are from Machias Seal Island, 1995–2011. Models were general linear models run using the “*nlme*” R package in the RStudio environment and include the terms Fledge Year and Sex as random effects.
(DOCX)

S6 Table Model averaged parameter estimates, unconditional standard errors, and relative likelihoods for the candidate model set evaluating the relationship between male and female Atlantic puffin adult bill depth and environmental conditions (i.e., SST anomaly, mean maximum air temperature, and prey quality fed to chicks) at Machias Seal Island during 1995–2011. Parameters in bold font are those that do not bound zero. Model averaging was completed using the “*MuMIn*” R package in the RStudio environment.
(DOCX)
