# Supplementary material for: The incredible shrinking puffin: Decreasing size and increasing proportional bill size of Atlantic puffins nesting at Machias Seal Island
Source: PLoS One. 2024 Jan 17;19(1):e0295946. doi: 10.1371/journal.pone.0295946 (PMC10793900; doi:10.1371/journal.pone.0295946)
Supplement: S1 Fig — Map generated from NOAA OI SST V2 High Resolution Dataset (https://psl.noaa.gov/mddb2/makePlot.html?variableID=156646). Arrows denote approximate location of each colony. (DOCX) [file pone.0295946.s001.docx]

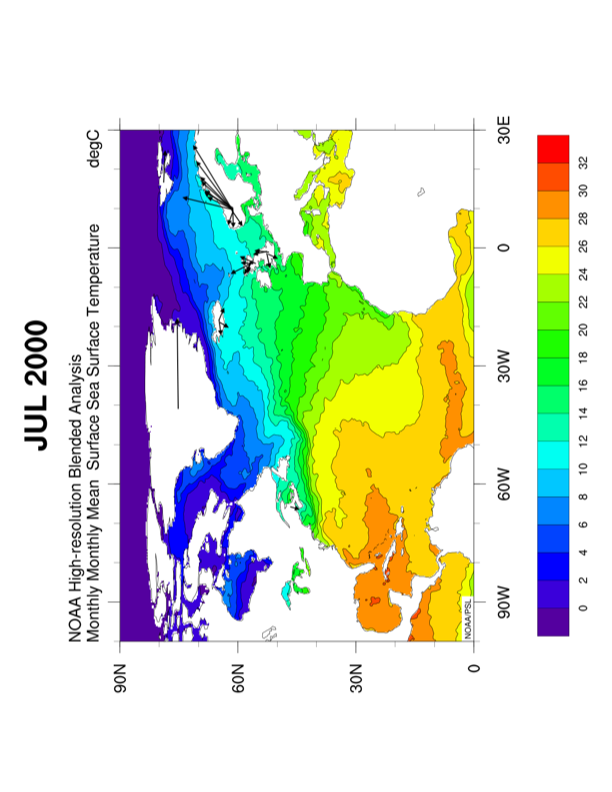


S1 Figure. Mean map monthly Sea Surface Temperature (SST; ^o^C) for the North Atlantic and Arctic oceans for July 2000. Map generated from NOAA OI SST V2 High Resolution Dataset ([https://psl.noaa.gov/mddb2/makePlot.html?variableID=156646](about:blank)). Arrows denote approximate location of each colony.
