# Supplementary material for: The incredible shrinking puffin: Decreasing size and increasing proportional bill size of Atlantic puffins nesting at Machias Seal Island
Source: PLoS One. 2024 Jan 17;19(1):e0295946. doi: 10.1371/journal.pone.0295946 (PMC10793900; doi:10.1371/journal.pone.0295946)
Supplement: S2 Fig — Data collated from A) Table 2 in Burnham et al. 2020 [68] and B) Appendix 1 in Harris and Wanless 2011 [53]. (DOCX) [file pone.0295946.s002.docx]

S2 Figure. Proportional bill size of the three Atlantic puffin (*Fratercula arctica*) subspecies (*F.a. arctica, grabae, and naumanni*) over their North Atlantic distribution. Data collated from A) Table 2 in Burnham et al. 2020 and B) Appendix 1 in Harris and Wanless 2011.
