# Supplementary material for: The incredible shrinking puffin: Decreasing size and increasing proportional bill size of Atlantic puffins nesting at Machias Seal Island
Source: PLoS One. 2024 Jan 17;19(1):e0295946. doi: 10.1371/journal.pone.0295946 (PMC10793900; doi:10.1371/journal.pone.0295946)
Supplement: S1 Table — All data are from Machias Seal Island, 1995–2011. Models were general linear models run using the “nlme” R package in the RStudio environment and include the term Fledge Year as a random effect. (DOCX) [file pone.0295946.s003.docx]

**S1 Table.** Candidate models assessing whether male and female Atlantic puffin adult head+bill (HB), wing chord (WC), ratio between bill depth and wing chord (BD:WC), and the ratio between bill area and wing chord (BA:WC) are a function of SST anomaly, mean maximum air temperature, and prey quality fed to chicks. All data are from Machias Seal Island, 1995 – 2011. Models were general linear models run using the “*nlme*” R package in the RStudio environment and include the term Fledge Year as a random effect.
