# Supplementary material for: The incredible shrinking puffin: Decreasing size and increasing proportional bill size of Atlantic puffins nesting at Machias Seal Island
Source: PLoS One. 2024 Jan 17;19(1):e0295946. doi: 10.1371/journal.pone.0295946 (PMC10793900; doi:10.1371/journal.pone.0295946)
Supplement: S2 Table — Parameters in bold font are those that do not bound zero. Model averaging was completed using the “MuMIn” R package in the RStudio environment. (DOCX) [file pone.0295946.s004.docx]

**S2 Table.** Model averaged parameter estimates, unconditional standard errors, and relative likelihoods for the candidate model sets evaluating the relationship between male and female Atlantic puffin adult head+bill, wing chord, ratio between bill depth and wing chord, and ratio between bill area and wing chord, and SST anomalies at Machias Seal Island during 1995 – 2011. Parameters in bold font are those that do not bound zero. Model averaging was completed using the “*MuMIn*” R package in the RStudio environment.
