# Supplementary material for: The incredible shrinking puffin: Decreasing size and increasing proportional bill size of Atlantic puffins nesting at Machias Seal Island
Source: PLoS One. 2024 Jan 17;19(1):e0295946. doi: 10.1371/journal.pone.0295946 (PMC10793900; doi:10.1371/journal.pone.0295946)
Supplement: S4 Table — Data from Appendix 1 Harris and Wanless 2011; sea surface temperature (SST) estimated from NOAA OI SST V2 High Resolution dataset for July 2020 (see Fig 1). (DOCX) [file pone.0295946.s006.docx]

**S4 Table.** Comparison of proportional bill size (straight bill length) to wing chord length of Atlantic puffins (*Fratercula arctica*). Data from Appendix 1 Harris and Wanless 2011; sea surface temperature (SST) estimated from NOAA OI SST V2 High Resolution dataset for July 2020 (see Figure 1).
