# Supplementary material for: The incredible shrinking puffin: Decreasing size and increasing proportional bill size of Atlantic puffins nesting at Machias Seal Island
Source: PLoS One. 2024 Jan 17;19(1):e0295946. doi: 10.1371/journal.pone.0295946 (PMC10793900; doi:10.1371/journal.pone.0295946)
Supplement: S6 Table — Parameters in bold font are those that do not bound zero. Model averaging was completed using the “MuMIn” R package in the RStudio environment. (DOCX) [file pone.0295946.s008.docx]

**S6 Table.** Model averaged parameter estimates, unconditional standard errors, and relative likelihoods for the candidate model set evaluating the relationship between male and female Atlantic puffin adult bill depth and environmental conditions (i.e., SST anomaly, mean maximum air temperature, and prey quality fed to chicks) at Machias Seal Island during 1995 – 2011. Parameters in bold font are those that do not bound zero. Model averaging was completed using the “*MuMIn*” R package in the RStudio environment.
